# Elements of Physiology

**Published:** 1839-01

**Authors:** 


### Art. XII.

1. *Elements of Physiology; being an Account of the Laws and Principles of the Animal Economy, especially in reference to the Constitution of Man.* By Thomas J. Aitkin, m.d. f.r.c.s.e. &c. &c.—*London*, 1838. 12mo. pp. 514.
2. *Popular Physiology; being a familar Explanation of the most interesting Facts connected with the Structure and Functions of Animals, and particularly of Man.* By Perceval B. Lord, m.b. m.r.c.s.—*London*, 1834. 12mo. pp. 500.
3. *Outlines of Human Physiology; designed for the use of the higher Classes in common Schools.* By George Hayward, m.d., Professor of Surgery in Harvard University. *Second Edition.—Boston* (*New England*), 1838. 12mo. pp. 222.

These three little volumes are all written with the very excellent object of giving to the young a general view of a science, which might have been expected to be the one most universally taught wherever education is afforded. The number of works which have lately issued from the press, designed to enforce those practical rules for the management of the body to which the study of physiology would of itself lead the intelligent mind, is a sufficient indication of the progress of enlightenment on this point. But we would have physiology and natural history studied by the young as a means of intellectual discipline; and we cannot account for the neglect with which the knowledge of the works of nature has been treated, whilst the learning of man has been exalted so highly. "Whilst we teach our children many tongues," we have heard it well remarked, "and enable them to understand many books, shall we leave them in ignorance of the great Book of Nature, which, to use the impressive language of Lord Bacon, 'is written in the only language that hath gone forth to all the ends of the earth, unaffected by the confusion of Babel.'" We perfectly accord, then, with the following sentiments expressed by Dr. Aitkin in his preface:

"In tracing the many curious contrivances which are exhibited in the organization of the human body, in perceiving the admirable adaptation of its parts to the performance of their various offices, and in viewing the whole series of its organs in their mutual relation and subserviency, one exercises the highest faculties of his mind, and acquires information that may be useful in life. Indeed, so obvious is the importance of an acquaintance with the bodily frame, that one, viewing the subject in a general sense, might naturally wonder why anatomy and physiology are not considered as indispensable elements of education."

We have been not a little surprised to meet, in a recent essay on Education, which professed to give the most enlightened view of the subject, with a passage implying that physiology, from its very nature, was totally unfit to be made a branch of instruction to the young. Now we cannot see why this should be; for, granting that there are certain parts of it which must be either omitted altogether or treated in a peculiar manner, there is nothing else, we conceive, that need prevent the teacher from entering as deeply into the science as he deems proper: and, on these subjects which are usually *tabooed* most sacredly, much information may, we are sure, be communicated, without the slightest invasion of even female delicacy, by a proper selection of topics. The great point *here* is to illustrate processes which every child knows to take place, without referring to those which, it is universally agreed, should be kept in the back-ground. What possible evil, we will ask, can result from tracing the development of the chick during incubation, and from showing the curious correspondence between the different stages of the evolution of its organs, and the permanent forms of the same in the lower animals? or in demonstrating the interesting process of fertilization in plants? or in explaining the process of reproduction in sponges and polypes? But, it may be objected, *any* allusion to subjects of this kind with the young will cause their thoughts to dwell upon what are at all times matters of sufficient curiosity. To this we would answer, that we would communicate such knowledge, and familiarize the mind to regard it as *not* of a mysterious character, *before* the time when the thoughts naturally tend to dwell upon it; and we cannot but regard the early inculcation of a taste for natural science as the best preservative against the injurious tendency of many works to which the young, either secretly or by permission, obtain access. To the female mind, in particular, the undisguised exhibition, in glowing colours, of the passion of love and its effects seems to us much more baneful than the scientific explanation of many physiological processes, which may be made both highly interesting and free from the remotest allusion to things which it is desirable not to name. For this purpose we prefer the use of purely scientific terms, without their popular explanations; and should rather, for example, speak of stamens and pistils, in describing the structure and actions of a flower, than of male and female organs. We know an instance in which a lecturer, who fully illustrated this department of vegetable physiology to an audience principally consisting of ladies, was afterwards thanked, by clergymen who had been his hearers, for having shown the possibility of combining a complete exposition of it with the most perfect purity of thought and language. That Dr. Lord's volume appears in the series of works published by the Christian Knowledge Society is a sufficient indication of the sentiments of that body upon the utility of the study of physiology as a branch of general education.

We have said more on this subject than it may be thought by some to require; but we believe that the errors to which we have alluded are shared nearly as much by our professional brethren as by the public at large. We must now say a few words of the respective characters of the volumes on which we have founded them.

We think that Dr. Aitkin could scarcely have been aware of the existence of the second work on our list when he undertook the first. They are so much alike in plan and in general style of execution, that it would be difficult to decide upon the superiority of either, each having its peculiar merits. Dr. Aitkin's volume strikes us as having a few redundancies, more fitted for the professional than the general reader: the latter, we think, will not be much benefited by the account of the doctor's sufferings from a wound received in dissection, which occupies two pages and a half, nor by various anatomical minutiæ of no immediate practical application. The history of the development of the human fœtus might also have been either omitted or modified with advantage, as in its present form it has no scientific value, and to the general reader can be of no real benefit. The author has not been able to free himself from a degree of technicality which will, we fear, embarrass and alarm many of his readers; and in this respect we think Dr. Lord's work decidedly superior, although it is deficient in many topics which Dr. Aitkin has treated well and fully. Both these treatises, however, are too full to serve as mere class-books, and are better adapted for the private reader. The third book on our list is one which appears to be well received in America with the former object; and is, from its more limited scope, better adapted to it. This, however, is somewhat too meager; and altogether omits references to the comparative structure of animals, which seems to us one of the most interesting departments of the subject. We have not yet seen anything in our language to equal the first part of the "Elémens de Zoologie" of M. Milne-Edwards; and we think that a concise treatise, similar to this in general plan, might be extensively used in our schools with great advantage to the rising generation.

The following case related by Dr. Aitkin will, we think, be interesting to many of our readers, from its character being exactly the converse of one which we formerly detailed. (Vol. IV. p. 500.)

"A youth fell from a wall ten feet high on a hard footpath, and was taken up insensible. Half an hour after, he was found in the same condition; blood flowed from the left ear, behind which, from external marks, it was evident the head had been struck. While he lay in a state of insensibility, with laborious breathing, the difference between the right and left side of the face was most striking: the right was thrown into a state of great agitation, and frequently affected with convulsive twitches, the eyelids contracted, the nostril dilated, and the angle of the mouth drawn up spasmodically on every inspiration. On the contrary, the left side remained in a state of perfect placidity. After his recovery from the more dangerous consequences, it was ascertained that the left side of the face had sustained permanent injury, which no means that were had recourse to had the effect of removing. His speech was also affected, having become hesitating and stammering. The unusual expression of his face and impediment of speech attracting the attention and ridicule of his thoughtless associates, he became morose in his temper and unsocial in his habits. He is now a very athletic young man, but still prefers solitude with his books, and is keenly alive to the peculiarities of his features and speech. Among those with whom he is in the habits of intimacy, and when he is off his guard, it is extremely curious to watch the changes of his countenance. If he laughs, it is only with one side of his face; when he frowns, the left side remains in a state of perfect indifference, as is the case whatever mental excitement he may be under at the time. *He possesses voluntary power over the muscles affected as completely as ever,* and the sensibility has never in the slightest degree been impaired. The commands of the will continue to be communicated to the muscles through the facial nerve, which received the shock, while the impulses of sympathy are no longer transmitted by it."

Cases exactly parallel to this and the one formerly quoted by us, but involving another class of sympathetic movements,—those of the chest in respiration,—will be found in the appendix to Sir C. Bell's volume on the Nerves. In some of these there has been paralysis of the respiratory muscles to the influence of the will, but their regular sympathetic movements have continued; whilst in others *these* have been interrupted, whilst the will has remained capable of producing contractions.

---

